# The Evolving Role of Eosinophils in Eosinophilic Esophagitis: Mechanisms, Crosstalk, and Therapeutic Perspectives

**DOI:** 10.3390/cells15131200

**Published:** 2026-07-01

**Authors:** Federico Caldart, Luisa Bertin, Annalisa Tortora, Alberto Barchi, Niccolò Seregni, Pierfrancesco Visaggi, Nicola De Bortoli, Marco Caminati, Marco Zurlo, Luca Frulloni, Edoardo Vincenzo Savarino

**Affiliations:** 1 Gastroenterology Unit, Department of Medicine, Verona University Hospital, Piazzale L.A. Scuro 9, 37134 Verona, Italy; annalisa.tortora@aovr.veneto.it (A.T.); luca.frulloni@univr.it (L.F.); 2 Department of Surgery, Oncology and Gastroenterology, University of Padua, Via Giustiniani 2, 35128 Padua, Italyedoardo.savarino@unipd.it (E.V.S.); 3 Gastroenterology Unit, Azienda Ospedale Università of Padua, Via Giustiniani 2, 35128 Padua, Italy; 4 Division of Gastroenterology and Gastrointestinal Endoscopy, IRCCS Ospedale San Raffaele, Via Olgettina 58, 20132 Milan, Italy; barchi.alberto@hsr.it (A.B.); seregni.niccolo@hsr.it (N.S.); 5 Faculty of Medicine, Università Vita-Salute San Raffaele, Via Olgettina 58, 20132 Milan, Italy; 6 Gastroenterology Unit, Department of Translational Research and New Technologies in Medicine and Surgery, University of Pisa, Lungarno Pacinotti 43, 56126 Pisa, Italynicola.debortoli@unipi.it (N.D.B.); 7 Digestive Endoscopy Unit, Department of General Surgery, Pisa University Hospital, Via Roma 67, 56126 Pisa, Italy; 8 Allergy Unit and Asthma Center, Department of Medicine, Verona Integrated University Hospital, Piazzale L.A. Scuro 9, 37134 Verona, Italy; marco.caminati@univr.it (M.C.);

**Keywords:** eosinophilic esophagitis, eosinophils, GERD

## Abstract

**Highlights:**

**What are the main findings?**

- Eosinophils are key orchestrators of EoE pathogenesis, driving epithelial barrier dysfunction, chronic type 2 inflammation, and progressive fibrostenotic remodeling through complex interactions with Th2 cytokines, mast cells, epithelial cells, and stromal pathways.
- Emerging evidence indicates that EoE extends beyond an eosinophil-driven disorder, involving a broader inflammatory network in which mast cells, Th2 lymphocytes, ILC2s, dendritic cells, and epithelial-derived alarmins contribute to disease persistence, fibrosis, and symptom generation.

**What are the implications of the main findings?**

- Therapeutic strategies focused exclusively on eosinophil depletion may be insufficient, as persistent symptoms and tissue remodeling can be sustained by mast cells, epithelial dysfunction, and upstream type 2 inflammatory pathways despite histologic eosinophil reduction.
- Future management of EoE should shift toward precision-medicine approaches that target multiple components of the type 2 inflammatory cascade, with early intervention aimed at preventing fibrosis, irreversible esophageal dysfunction, and long-term disease progression.

**Abstract:**

Introduction: Eosinophilic esophagitis (EoE) is a chronic immune-mediated esophageal disease characterized by eosinophilic infiltration, epithelial barrier dysfunction, and progressive tissue remodeling. Increasing evidence identifies eosinophils as central drivers of inflammation and fibrosis, linking EoE to type 2 immune responses and allergic disorders. However, the molecular mechanisms underlying eosinophil-mediated esophageal damage and their interaction with gastroesophageal reflux disease (GERD) remain incompletely understood. Material and Methods: A comprehensive narrative review of the current literature was conducted, focusing on studies investigating eosinophil biology, inflammatory signaling pathways, epithelial remodeling, fibrosis, and therapeutic targets in EoE. Clinical, translational, and experimental studies evaluating the association between EoE, GERD, and allergic comorbidities were critically analyzed. Results: Available evidence demonstrates that eosinophils actively contribute to EoE pathogenesis through the release of cytotoxic granule proteins, cytokines, chemokines, and lipid mediators, leading to chronic inflammation and fibrostenotic remodeling. Dysregulation of type 2 cytokines, particularly IL-4, IL-5, and IL-13, plays a pivotal role in disease progression and immune cell recruitment. Significant overlap between EoE and GERD suggests shared inflammatory mechanisms and diagnostic challenges. Furthermore, EoE frequently coexists with systemic allergic diseases, supporting the concept of a broader atopic inflammatory phenotype. Emerging biologic therapies targeting eosinophilic and type 2 inflammatory pathways have shown promising efficacy in reducing symptoms and histologic activity. Conclusions: Eosinophils represent key regulators of EoE pathobiology and constitute promising biomarkers and therapeutic targets. A deeper understanding of eosinophil-driven inflammatory networks may improve diagnostic accuracy, patient stratification, and the development of personalized therapeutic strategies for EoE and related esophageal inflammatory disorders.

## 1. Introduction

Eosinophilic esophagitis (EoE) is a chronic, type-2 helper T-cell (Th2)-mediated disease, characterized by a predominant eosinophilic infiltrate, that leads to symptoms of esophageal dysfunction [1,2]. The prevalence and incidence of EoE have been steadily increasing. They are going to double in ten years, as a result of increased true prevalence, a pattern similarly observed in other atopic diseases, such as atopic dermatitis, food allergies, allergic rhinitis, and asthma [3,4], and raised disease awareness with more frequent esophageal biopsies performed during esophagogastroduodenoscopy (EGD). Data from a population-based study conducted in Spain between 200 and 2024 report an average incidence of 9.41–11.49 cases/100,000 inhabitants/year and a prevalence in 2024 of 194.73–251.39 cases/100,000 inhabitants [5]. EoE is part of Th2-mediated disorders, which include atopic dermatitis (AD), food allergies (FA), allergic rhinitis (AR), and asthma [6]. In this context, eosinophils play a central role in disease pathogenesis, acting as key drivers of inflammation and fibrostenotic progression. The accumulation and degranulation of eosinophils within the esophageal epithelial barrier are considered among the main mechanisms leading to mucosal damage, aero- and food-antigen sensitization, and alterations in esophageal motility [4,7,8,9,10]. Recent data, however, suggest that EoE is more than an eosinophilic-dependent disease. As proinflammatory cells, eosinophils are part of the Th2 Cascade, which, along with other effector cells such as basophils and mast cells, contributes to the clinical manifestations of EoE [11]. Clinical trials with eosinophil-depleting agents (e.g., mepolizumab, reslizumab, benralizumab, and lirentelimab) achieve substantial histologic improvement. Still, they have generally failed to provide meaningful symptomatic relief, suggesting that eosinophil depletion alone is insufficient [11,12,13]. This evidence is consistent, for instance, with data from the “MESSINA trial” [14], where benralizumab, a monoclonal antibody directed against the IL-5 receptor, reduces eosinophil counts but does not resolve dysphagia. This trial, along with previous studies on anti-IL-5 biologics, raises questions about the pathological relevance of targeting eosinophils exclusively in the treatment of EoE [15]. This narrative review summarizes the complex role of eosinophils in EoE, focusing on their contribution to disease pathogenesis and their relevance as therapeutic targets.

## 2. Role of Eosinophils in Inflammation in EoE

Eosinophils are granulocytic, myeloid-derived cells that develop in the bone marrow under the influence of specific transcription factors and cytokines, including interleukin-3 (IL-3), granulocyte-macrophage colony-stimulating factor (GM-CSF), and IL-5 [16,17]. They serve as key effector cells in EoE, recruited through a Th2-mediated inflammatory cascade involving multiple cytokines, chemokines, and cellular interactions, ultimately leading to tissue damage, remodeling, and fibrosis [2,18]. This pathway is in common with other Th2-mediated disorders, such as asthma, AR, AD, and Chronic Rhinosinusitis with Nasal Polyps (CRSwNP), as part of the “atopic march” [9,19,20].

### 2.1. Migration, Maturation, and Activation of the Eosinophils

In healthy individuals, eosinophilic infiltration is absent in the esophagus. Their migration into the bloodstream, mediated in part by eotaxins, and subsequent recruitment to peripheral tissues are closely linked to their maturation [16,17,19]. Mature eosinophil granules store cytokines, chemokines, growth factors, and lipid mediators that further modulate immune responses. IL-1, IL-25, IL-33, thymic stromal lymphopoietin (TSLP), alarmins (e.g., uric acid, ATP, HMGB1, and S100 proteins) promote the inflammatory Th-2 cascade in the epithelial cells [21]. The production of IL-5, IL-4, IL-13, and eotaxin (CCL26) by dendritic cells (DCs) and Th2 cells contributes to eosinophil migration and activation in the esophageal mucosa [17,22,23,24,25]. Notably, the interaction between IL-5 and its high-affinity receptor (IL-5Rα) triggers intracellular signaling pathways, including the Janus kinase-signal transducer and activator of transcription (JAK-STAT) pathway, which regulates eosinophil proliferation, differentiation, and survival [16,26]. Additionally, IL-4 contributes to eosinophil chemotaxis and promotes the expression of adhesion molecules by activating the Th2 pathway [27,28,29]. Given the pivotal role of IL-5 and CCL26 in eosinophil maturation, activation, and trafficking, IL-13-induced upregulation of CCL26 is particularly relevant and has been shown to correlate with disease activity in EoE, with marked overexpression in the EoE transcriptome (up to 53-fold) [19,28,30]. Furthermore, epithelial-derived alarmins, including TSLP and IL-33, are rapidly released in response to tissue injury, further amplifying type 2 inflammation. Emerging evidence, largely derived from flow cytometry studies, has begun to characterize distinct phenotypic features of circulating eosinophils that may facilitate their homing to the inflamed esophagus. In patients with EoE, peripheral blood eosinophils exhibit increased expression of surface molecules involved in chemotaxis, adhesion, and activation, including the low-affinity IgE receptor CD23, intercellular adhesion molecule-1 (ICAM-1/CD54) [31,32], the CC chemokine receptor CCR3 (the principal receptor for eotaxins) [33], integrin CD11c, and the prostaglandin D2 receptor CRTH2 [31,32], as well as elevated FOXP3 mRNA expression [31]. Several of these molecules have been proposed as potential therapeutic targets in EoE. Based on differential expression of these inflammatory pathways, Shoda et al. [34] identified three distinct molecular endotypes of EoE (EoEe1–3) using the Eosinophilic Esophagitis diagnostic panel (EDP), comprising 96 informative transcripts. EoEe1 is characterized by a predominantly normal-appearing esophagus (risk ratio [RR] 3.27), a lower likelihood of prior esophageal dilation (RR 0.27), and relatively mild histologic, endoscopic, and molecular features. In contrast, EoEe2 exhibits a highly inflammatory and steroid-refractory phenotype (RR 2.77, 95% CI 1.11–6.95; *p* = 0.0376), with elevated expression of proinflammatory cytokines and steroid-responsive genes. EoEe3 is associated with adult-onset disease and a narrow-caliber esophagus (RR 7.98, 95% CI 1.84–34.64; *p* = 0.0013), and is characterized by the most severe endoscopic and histologic findings, along with reduced expression of epithelial differentiation genes.

### 2.2. Role of the Eosinophils in the Disruption of the Esophageal Epithelial Barrier

EoE-associated inflammation arises from a complex interplay among genetic susceptibility, environmental factors, the microbiome, and eosinophilic infiltration, which is linked to disruption of the esophageal epithelial barrier [35]. Multiple genetic polymorphisms in inflammatory cytokine genes have been studied to identify susceptibility genes associated with an increased risk of EoE [35,36,37,38,39,40]. More than 20 genes have been identified, including the TSLP gene at 5q22, the calpain 14 gene on chr2p23.1, and c11orf30 [35,36,37,38,39,40]. In genetically predisposed subjects, the Th2-mediated cascade is induced by exposure to food- or aeroallergens via IL-4 and IL-13 pathways, contributing to damage to esophageal epithelial cells. The epithelial barrier is highly compromised in active EoE by the eosinophils [40]. Many studies carried out with electron microscopy [41] and impedance [42] showed a decrease in tight junctions and their components (e.g., decrease in claudin-1, claudin-4, claudin-7, occludin, and zonula occludin-1 proteins, E-cadherin, desmoglein-1, involucrin, and filaggrin expression) in the esophageal mucosa, with dilated intercellular spaces. In addition, eosinophils typically follow a density gradient toward the luminal surface, with the highest concentration in the most superficial layers, which are directly exposed to ingested allergens [43]. Notably, eosinophils often aggregate within these layers, forming characteristic microabscesses [44,45]. Recently, the reduction of serine protease inhibitor, kazal type 7 (SPINK7), an antiprotease involved in the normal differentiation of the esophageal epithelium, has been linked to epithelial changes in active EoE [46,47]. Silencing or loss of SPINK7 promoted the production of proinflammatory cytokines, including TSLP, and the augmentation of the activity of the urokinase plasminogen-type activator (uPA), leading to uPA receptor-dependent eosinophil activation [46,48]. In addition, the recent identification of the transcription factor, ovo-like transcriptional repressor 1 (OVOL1), has been linked to the regulation of SPINK7 promoter activity [48]. Indeed, higher OVOL1 levels increase SPINK7 expression, whereas reduced OVOL1 levels decrease SPINK7 expression, compromise epithelial barrier integrity, and enhance TSLP production. Stimulation with IL-13, mediated by calpain-14, prevents OVOL1 from moving into the nucleus and increases its protein degradation. In human esophageal biopsy samples, OVOL1 levels are positively associated with SPINK7 expression and tend to decline as EoE disease activity increases [48]. In conclusion, eosinophil-mediated disruption of epithelial barrier function is a central component of the pathogenesis of EoE, and specific EoE treatments, both directly and indirectly, improve the integrity of the esophageal mucosa against aero- and food-allergens.

## 3. Role of Eosinophils in Fibrosis and Tissue Remodeling in EoE

EoE is now recognized as a progressive fibrostenotic disease in which chronic type 2 inflammation drives tissue remodeling with potentially irreversible structural and functional consequences [49,50,51]. The transition from inflammatory to fibrostenotic phenotypes represents a critical determinant of long-term morbidity, as stricture prevalence increases from 17.2% in patients with diagnostic delays of 0–2 years to 70.8% in those with delays exceeding 20 years [52]. Notably, each additional untreated year increases the odds of stricture by approximately 26% [53]. Understanding the molecular mechanisms underlying eosinophil-mediated fibrosis is essential for developing targeted therapeutic interventions that can prevent or reverse esophageal remodeling [11,12,54,55,56].

### 3.1. Molecular Mechanisms of Fibrogenesis

**Figure 1:**
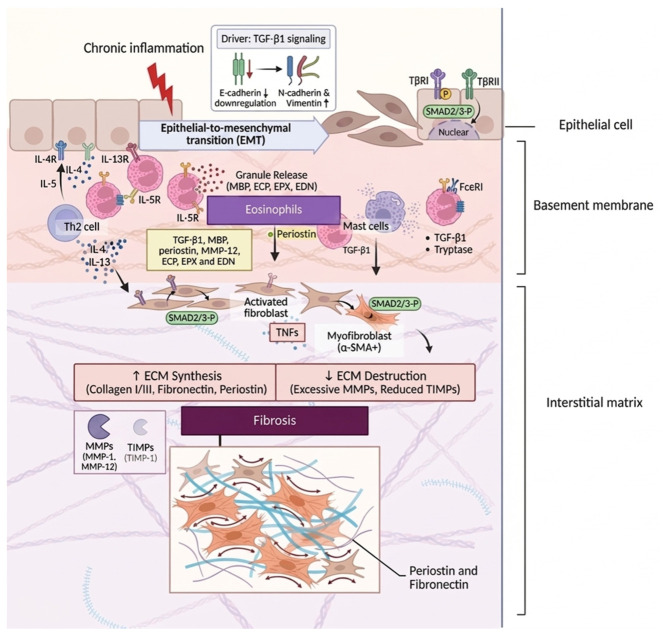
Eosinophil-related fibrosis.

Transforming growth factor-beta 1 (TGF-β1) serves as the master regulator of fibrosis in EoE, orchestrating multiple downstream pathways through canonical SMAD2/3 signaling [57,58,59]. This cytokine, produced abundantly by eosinophils and mast cells infiltrating the esophageal mucosa, drives epithelial–mesenchymal transition (EMT), fibroblast activation, extracellular matrix (ECM) deposition, and smooth muscle dysfunction [60,61]. The type 2 cytokine triad of IL-13, IL-5, and IL-4 synergistically amplifies TGF-beta effects, with IL-13 particularly implicated in promoting fibroblast-to-myofibroblast differentiation and ECM production [62,63,64]. Experiments using *Krt14Cre*; *Il13ra1^fl/fl^* mice, in which epithelial type II IL-4 receptor is selectively deleted, demonstrate limited oxazolone-induced lamina propria thickening, indicating that epithelial signaling downstream of IL-4/IL-13 contributes to stromal fibrosis [65]. Recent single-cell RNA sequencing studies have revealed that esophageal eosinophils exist as two distinct populations: a minority resembling peripheral blood eosinophils and a predominant, activated population expressing diverse sensing receptors and inflammatory mediators, capable of interacting with multiple cell types [66]. Figure 1 illustrates the mechanisms behind eosinophil-related fibrosis.

Eosinophil-derived mediators contribute directly to tissue injury and remodeling. Major basic protein (MBP) induces epithelial damage and basal zone hyperplasia through upregulation of fibroblast growth factor-9 (FGF-9) [67]. Eosinophils also produce periostin, an ECM protein that promotes fibroblast proliferation and collagen deposition, as well as matrix metalloproteinase-12 (MMP-12), which contributes to tissue remodeling [68,69,70]. Studies using eosinophil-deficient murine models have demonstrated that eosinophils are essential for allergen-induced esophageal fibrosis, as mice lacking eosinophils or IL-5 are protected from subepithelial collagen accumulation [71,72]. Recent investigations have identified mesenchymal progenitor cell-like (MPCL) fibroblasts in EoE biopsies that exhibit pathogenic regenerative gene expression programs that drive tissue dysfunction [73,74]. These fibroblasts demonstrate enrichment in extracellular matrix structural constituents and in osteogenesis pathways, suggesting a maladaptive wound-healing response. Emerging evidence implicates bidirectional crosstalk between esophageal epithelial cells and underlying stromal cells as a key driver of lamina propria remodeling in EoE [75,76]. Fibroblast-derived tumor necrosis factor-alpha (TNF-α) induces esophageal epithelial cells to produce lysyl oxidase (LOX), a collagen crosslinking enzyme whose expression is significantly elevated in biopsies from patients with fibrostenotic EoE [77].

Additionally, fibroblast-derived TNF-α and IL-1β drive EMT in esophageal keratinocytes, leading to epithelial cells acquiring the functional characteristics of activated myofibroblasts, including collagen production, enhanced migration, and contractility [58]. Evidence of EMT, characterized by reduced epithelial cytokeratin expression with concomitant upregulation of vimentin, has been documented in EoE patient biopsies where it positively correlates with eosinophil counts, TGF-β immunostaining, and subepithelial fibrosis [78]. A landmark 2025 study identified MPCL fibroblasts in EoE with dysfunctional tissue regenerative programs [79]. These pathogenic fibroblasts demonstrate intact chondrogenic and osteogenic differentiation but markedly reduced adipogenic differentiation capacity, suggesting a shift toward the generation of rigid tissue that may underlie esophageal stiffening. Single-cell RNA sequencing revealed that EoE MPCL fibroblasts display proinflammatory and pro-rigidity transcriptional programs enriched for interferon response and mitochondrial activity pathways. Critically, EoE fibroblasts showed significantly decreased surface CD73 expression and enzymatic activity compared to healthy controls. CD73 is a 5′-nucleotidase that converts extracellular adenosine monophosphate to anti-inflammatory adenosine; its deficiency drives the pathogenic phenotype. Adenosine repletion rescued EoE fibroblast migration and tissue-regenerative dysfunctions, while CD73 inhibition in healthy fibroblasts phenocopied EoE abnormalities. These findings identify aberrant extracellular ATP handling as a novel therapeutic target [80,81].

### 3.2. Histological Components of Tissue Remodeling

Esophageal remodeling in EoE encompasses distinct epithelial and subepithelial changes that collectively impair organ function. In the epithelium, basal zone hyperplasia reflects active proliferation of progenitor cells, driven by MBP and FGF-9, while EMT results in loss of epithelial characteristics and acquisition of mesenchymal features, including vimentin expression [73,74]. The EoE Histologic Scoring System (EoEHSS) captures these changes by assessing eight features beyond simple eosinophil enumeration, providing superior patient discrimination compared to peak eosinophil counts [82,83]. Barrier dysfunction represents an early pathological event, with downregulation of desmoglein-1 and dilated intercellular spaces reflecting compromised epithelial integrity that may persist even during histologic remission [84]. Subepithelial remodeling includes lamina propria fibrosis characterized by collagen deposition, fibroblast accumulation, and smooth muscle hypertrophy. TGF-β induces smooth muscle proliferation and enhances contractility by altering phospholamban-mediated calcium handling, contributing to the esophageal dysmotility observed in 25–83% of EoE patients [85,86]. Importantly, assessing lamina propria fibrosis from routine biopsies is challenging, as the average esophageal biopsy depth of 0.5–1 mm samples only a fraction of the full 10 mm wall thickness [87]. Studies using large-capacity forceps found that only 55% of biopsies included subepithelial tissue, even with optimal positioning [88]. This limitation has driven interest in functional assessments such as the endoscopic Functional Lumen Imaging Probe (EndoFLIP) as complementary measures of fibrosis severity.

### 3.3. Clinical Consequences and Assessment of Esophageal Fibrosis

The fibrostenotic transformation of EoE manifests clinically as progressive dysphagia, food impaction, and stricture formation. The Swiss EoE Cohort demonstrated that the prevalence of fibrotic features increases from 46.5% in patients with less than 2 years of diagnostic delay to 87.5% in those with more than 20 years of diagnostic delay [89]. Care gaps exceeding 2 years are associated with a 37% probability of developing severe fibrotic features, while maintenance therapy provides 88% risk reduction for repeat esophageal dilations [90]. However, recent data challenge assumptions about fibrostenosis definitions and progression. A 2025 systematic review analyzing 230 studies found substantial variability in how fibrostenosis is defined, with four different categorical approaches: structural findings (88.7%), histology (37%), functional parameters (6.5%), and biomarkers (3%) [91]. Interobserver agreement for endoscopic fibrotic features remains poor, with kappa values of only 0.40 for rings and 0.52 for strictures. EndoFLIP technology has emerged as a valuable tool for assessing esophageal mechanical properties. A distensibility index less than 4.5 mm^2^/mmHg defines esophageal rigidity in pediatric EoE patients aged 9 years and older, with distensibility inversely correlating with EoEHSS grade scores [92,93]. EndoFLIP demonstrates superior sensitivity for detecting fibrostenotic alterations compared to standard endoscopy, which has only 14.7% sensitivity for detecting a narrowed esophagus [94]. A physiomechanical classification system based on distensibility metrics and esophageal motility patterns has established seven distinct EoE categories ranging from preserved function to severe fibrostenosis with absent muscular reactivity [95]. Importantly, the first longitudinal prospective pediatric study demonstrated that histologic response is associated with improved esophageal distensibility over time, providing crucial evidence that disease control during childhood can positively impact mechanical function [96].

## 4. Eosinophils in EoE and Gastroesophageal Reflux Disease (GERD)

In the 1970s and 1980s, it was a prevailing notion that intraepithelial eosinophils in esophageal biopsies were a hallmark of GERD, resulting from chronic acid exposure, esophageal injury, and subsequent chronic inflammation [97]. Then, in 1993, Attwood et al. [98] reported a case series of 12 adult patients with increased intraepithelial esophageal eosinophils (>20 eos/HPF) and dysphagia in the absence of GERD based on upper endoscopy and pH testing. In 1994, Straumann et al. [99] further characterized this entity as esophageal eosinophilia with dysphagia in another series of 10 adults and named it “Idiopathic EoE”. In 2006, Ngo et al. [100] introduced the concept of proton pump inhibitor-responsive esophageal eosinophilia (PPI-REE), as PPIs, historically considered drugs for GERD [101], were shown to reduce mucosal eosinophil infiltration in patients with esophageal eosinophilia. Since then, several studies have shown that PPI-REE is a phenotype of EoE that responds to PPIs, as evidenced by similar clinical, endoscopic, histologic, and molecular features [1,2,102,103]. In addition, PPIs have been shown to reduce esophageal eosinophil burden by inhibiting Eotaxin-3-mediated eosinophil recruitment in the esophagus, thereby providing an acid-independent mechanism of action in EoE [104]. Although EoE is currently accepted as a distinct clinical entity separate from GERD, the relatively high prevalence of GERD in the general population (around 20% in Western countries) [105,106] means that a considerable number of patients with EoE would be expected to have coexisting GERD purely by coincidence [102]. However, because both conditions are associated with esophageal eosinophilia, distinguishing between them can be challenging. However, the clinical need to formally distinguish the two conditions has led to the establishment of a standardized histologic cut-off of eosinophils/hpf to distinguish GERD from EoE. In this regard, eosinophilic infiltration is typically mild in GERD (<7–10 eos/hpf) and predominantly confined to the distal esophagus, reflecting injury from acid exposure [107]. Pediatric studies further supported lower eosinophilic infiltrates in GERD compared to EoE, showing that ≥20 eos/hpf correlated with normal acid exposure. In contrast, lower eosinophil counts (<5 eos/hpf) were more consistent with abnormal acid exposure on ambulatory reflux monitoring, reinforcing the idea that mild eosinophilia could be secondary to acid-induced inflammation [108]. Additionally, a cut-off of >7 eos/hpf has been shown to predict failure of anti-reflux therapy, suggesting that intermediate eosinophilia may reflect non-GERD inflammatory processes [109]. Based on available evidence, from a purely histological standpoint, current international guidelines define EoE as the presence of at least 15 eos/hpf (or 60 eos/mm^2^) in at least one esophageal biopsy in the appropriate clinical context [1,2,103,110]. It must be emphasized, however, that the definition of EoE based on eosinophils/hpf alone may be too simplistic. Beyond eosinophil counts, important differences exist in other inflammatory mediators and immune pathways between GERD and EoE. In EoE, additional findings such as eosinophilic microabscesses, surface layering of eosinophils, eosinophil degranulation, and subepithelial fibrosis are more common and reflect ongoing immune-mediated tissue injury and remodeling [83,108]. Beyond histology, there is evidence supporting that EoE is a systemic, rather than an esophageal-restricted, disease. In this regard, it has been shown that peripheral blood eosinophils, as well as non-eosinophil-derived mediators, can distinguish EoE from non-EoE dysphagia, including GERD [111,112]. In addition, EoE is characterized by a type 2-driven immune response, involving cytokines and chemokines that recruit and activate eosinophils, promoting their survival and activation, thereby contributing to sustained inflammation and tissue remodeling. These mediators are much more prominent in EoE than in GERD and reflect an antigen-driven immune process rather than acid injury. GERD-related inflammation is primarily driven by chemical injury from acid and bile, leading to activation of epithelial stress responses and recruitment of inflammatory cells through nonspecific pathways. These include upregulation of adhesion molecules, such as vascular cell adhesion molecule (VCAM), and the release of chemotactic factors, including IL-8 and other nonspecific inflammatory mediators [113,114]. However, these mediators lack specificity and may reflect general epithelial injury rather than a defined immune response. Overall, eosinophils play a role in both EoE and GERD. However, the two conditions differ fundamentally in pathogenesis, with GERD driven by acid-mediated injury and EoE representing a type 2 immune-driven inflammatory disorder. Although histologic overlap exists, defined eosinophil thresholds and additional inflammatory and molecular features help distinguish the two conditions.

## 5. Eosinophil-Derived Mediators and Cytokines in the Pathogenesis of EoE

Cytokines and eosinophil-derived mediators play a central role in both the pathogenesis and natural history of EoE. This immune-mediated disorder is characterized by allergen-driven Th2 inflammation occurring in the context of an intrinsically or secondarily impaired esophageal barrier. A key pathogenic mechanism is IL-13-induced eotaxin-3 production, which promotes eosinophil recruitment [9,19,115,116]. In this context, eosinophils act as central effector cells in the inflammatory cascade, actively contributing to disease progression by releasing cytotoxic granule proteins, cytokines, and lipid mediators [117]. While Th2 lymphocytes and mast cells represent the primary sources of type 2 cytokines, eosinophils further amplify local inflammatory responses by producing their own mediators via autocrine and paracrine mechanisms [60,118,119]. Among the key Th2-associated cytokines involved in EoE pathogenesis, IL-5 plays a central role in maintaining tissue eosinophilia by promoting eosinophil survival, activation, and responsiveness to chemotactic signals, thus supporting their persistence within esophageal tissue [71,120]. Similarly, IL-13, primarily produced by Th2 cells, acts as a key upstream regulator of EoE by driving epithelial activation, inducing eotaxin-3 (CCL26) expression, and promoting a disease-specific transcriptional program associated with barrier dysfunction [115,121,122]. Eotaxin-3 (CCL26), induced by IL-13, plays a critical role in eosinophil recruitment via CCR3 signaling, serving as a key link between epithelial activation and eosinophilic inflammation. IL-4 exerts a complementary role by signaling through the shared IL-4Rα receptor, thereby enhancing IL-13-mediated effects on epithelial activation and eotaxin-3 production; the pathogenic relevance of this pathway is underscored by the clinical efficacy of IL-4/IL-13 blockade with dupilumab [123,124,125]. Epithelial-derived alarmins such as TSLP further amplify type 2 immune responses. Beyond inflammatory cytokines, pathways involved in tissue remodeling also play a critical role in disease progression. In this context, TGF-β1 plays a distinct role in EoE by linking chronic inflammation to tissue remodeling. Produced by eosinophils, mast cells, and epithelial cells, TGF-β1 promotes fibroblast activation and ECM deposition, contributing to the progression of fibrostenotic disease [126]. In EoE, increased TGF- β1 expression is associated with activation of SMAD2/3 signaling in the mucosa, driving fibrotic responses [127]. This includes enhanced production of ECM components, such as fibronectin and collagen I, by mesenchymal cells, as well as processes such as epithelial-to-mesenchymal transition [127]. This highlights a shift from inflammation to structural remodeling of the esophageal wall. In addition to cytokine-driven mechanisms, eosinophil-derived lipid mediators contribute to the amplification of inflammation in EoE. Key molecules such as platelet-activating factor, leukotriene B4, cysteinyl leukotrienes, and prostaglandin D2 regulate eosinophil recruitment, activation, and survival. LTB4 acts as a chemoattractant, while cysteinyl leukotrienes promote adhesion and autocrine/paracrine signaling pathways [128]. PAF and PGD2 strongly induce chemotaxis and enhance lipid body-dependent LTC4 synthesis [128,129,130]. Moreover, lipid mediators interact with cytokines and chemokines, creating a feed-forward inflammatory loop and contributing to epithelial barrier dysfunction in EoE [128]. Upon recruitment to the esophageal tissue, eosinophils undergo extensive degranulation, often via cytolysis, resulting in the extracellular release of granule contents [131]. As a result, eosinophils undergo structural disruption, and tissue damage can persist even in the absence of clearly identifiable intact cells on histological examination [132]. The extracellular deposition of eosinophil granule proteins—including major basic protein-1 (MBP-1), eosinophil cationic protein (ECP), eosinophil peroxidase (EPO), and eosinophil-derived neurotoxin (EDN)—mediates direct cytotoxic and oxidative tissue injury, thereby contributing to sustained epithelial damage [133]. Among these, MBP-1 plays a key role in epithelial injury by inducing membrane permeabilization via pore formation in lipid bilayers, thereby disrupting cellular ionic homeostasis [134]. In parallel, ECP and EPO further amplify tissue damage, with ECP forming non-selective membrane pores that facilitate cytotoxic effects and contribute to tissue remodeling, fibrosis, and hypertrophy. At the same time, EPO promotes oxidative tissue injury by generating reactive species [135,136]. Rather than acting simply as terminal effector cells, eosinophils can be viewed as active regulators of inflammation in EoE, integrating signals from cytokines, chemokines, and lipid mediators to sustain tissue damage. The interaction between eosinophil-derived mediators and epithelial dysfunction underlines the complexity of the disease, which extends beyond a purely Th2-driven process. A clearer understanding of these interconnected mechanisms has already improved the development of targeted therapies and will continue to guide future approaches to control eosinophil-driven inflammation.

## 6. Crosstalk Between Eosinophils and Other Cell Types in EoE

In addition to eosinophils, other immune cells are increasingly recognized as important contributors to EoE pathogenesis. Mast cells are tissue-resident immune cells that play an important role in allergic inflammatory responses. Under homeostatic conditions, mast cells are normally present within the esophageal mucosa and are predominantly localized in the lamina propria [28]. During active EoE, mast cells infiltrate and proliferate within the esophageal epithelium, where they become activated and degranulate. Several clinical studies have shown that mast cell numbers correlate with patient-reported pain symptoms [28,60,118,137,138]. Notably, a clinical trial investigating anti-IL-5 therapy in children with EoE demonstrated that pain severity was associated with esophageal mast cell levels rather than eosinophil counts. More recent findings have confirmed this association and further suggest that mast cells may interact with TRPV1 expression in the esophagus, an ion channel involved in the detection of painful stimuli by sensory neurons [139]. This interaction may contribute to the link between mast cells and pain in EoE. Importantly, mast cell infiltration within the epithelium may persist despite treatment-induced resolution of tissue eosinophilia. In addition, mast cell levels have been associated with persistent symptoms and ongoing endoscopic abnormalities, such as furrows and rings, as well as histologic alterations, including basal zone hyperplasia and dilated intercellular spaces [66,138,140]. Furthermore, mast cells produce IL-13 and eosinophil-activating cytokines, including IL-3, IL-5, and GM-CSF, thereby promoting eosinophil recruitment and survival [66]. T lymphocytes (especially CD4+ TH2 cells) are key drivers of eosinophilic inflammation. During active disease, these cells accumulate within the esophageal mucosa and secrete type 2 cytokines, including IL-4, IL-5, and IL-13, which support eosinophil recruitment, activation, and persistence in the tissue [43,141,142]. The expansion of pathogenic memory TH2 cells has been strongly associated with the degree of esophageal eosinophilia. TH2 cells also contribute to inflammation by producing prostaglandin D2 (PGD2), a mediator that acts via the CRTH2 receptor expressed on eosinophils and other type 2 immune cells [28,129,143,144,145]. Activation of this pathway promotes further eosinophil migration and activation, thereby amplifying the inflammatory response. Through these mechanisms, TH2 lymphocytes play a major role in maintaining chronic eosinophilic inflammation and epithelial dysfunction in EoE. Additional immune cells implicated in eosinophilic esophagitis (EoE) include basophils, type 2 innate lymphoid cells (ILC2s), and dendritic cells (DCs), all of which may contribute to eosinophilic inflammation. Basophils are increased in both blood and esophageal tissue during active EoE, and experimental models suggest that their activity may be linked to a TSLP-dependent inflammatory pathway [146,147,148,149]. ILC2s are activated by epithelial-derived alarmins such as IL-33 and TSLP and produce large amounts of type 2 cytokines, including IL-5 and IL-13, which promote eosinophil recruitment and activation [149,150]. Increased levels of esophageal ILC2s have been strongly associated with tissue eosinophilia in active EoE [149]. DCs are also expanded in the esophageal mucosa during EoE and may contribute to disease progression by promoting TH2 polarization through TSLP- and IL-33-mediated signaling, thereby indirectly sustaining eosinophilic inflammation [28,151].

## 7. Eosinophils in EoE and Associated Allergic Diseases

Epithelial barrier dysfunction is now recognized as a central component of the pathogenesis of EoE, asthma, and atopic dermatitis (AD), all of which are characterized by Th2 Inflammation. Although these conditions share common immunological features, the relative contributions of epithelial barrier impairment and Th2 immune dysregulation vary considerably both across diseases and among individual patients [152]. Despite being a histopathological hallmark of EoE, peripheral blood eosinophil counts do not reliably correlate with oesophageal eosinophilia or disease activity, unlike in asthma [153,154], as suggested by the limited efficacy of anti-IL-5-targeted therapies in EoE. Instead, markers of eosinophil activation, such as tissue degranulation, appear to correlate more closely with disease severity, including fibrosis [153]. Notably, circulating eosinophils in allergic conditions often lack morphological signs of activation, suggesting that tissue-specific activation rather than systemic eosinophilia is more relevant to disease pathophysiology. Furthermore, eosinophils may persist within the oesophageal epithelium even in clinical remission, indicating ongoing subclinical inflammation [153,155]. Although EoE, asthma, and AD share this Th2-driven inflammatory axis, disease-specific differences are evident. EoE and AD, in particular, share features of epithelial barrier dysfunction, including impaired tight junction integrity and reduced expression of structural proteins such as filaggrin and claudins [156,157]. These conditions are also linked through the “skin-gut axis” and are part of the atopic march, alongside asthma, allergic rhinitis, and food allergy. Indeed, these comorbidities frequently coexist, affecting more than half of patients. Therapeutically, this overlap is reflected in the efficacy of agents such as corticosteroids and the IL-4 receptor α antagonist dupilumab, which targets both IL-4 and IL-13 signaling pathways and improves epithelial barrier function across multiple Th2-mediated diseases [152,158]. EoE is also frequently associated with allergic rhinitis and food allergy [147,148,149]. In allergic rhinitis, aeroallergens are thought to contribute to esophageal inflammation, potentially explaining the seasonal exacerbation of EoE symptoms, even in treated patients [148,149]. Both systemic and local underlying mechanisms have been hypothesized. In sensitized individuals, the immune response triggered by allergen exposure upregulates systemic atopic inflammation, leading to eosinophil migration into the airways and esophagus [149]. In terms of local mechanisms, direct contact between pollen and the oesophageal mucosa, due to its poor integrity, seems to favor on-site inflammation [149]. From this perspective, the role of immunotherapy with inhalant allergens remains controversial, and the available evidence is limited. On one hand, some reports describe the onset or relapse of EoE symptoms in patients undergoing allergen immunotherapy. On the other hand, it is plausible that reducing individual sensitivity to airborne allergens could help restore epithelial function, as suggested by other studies [148]. The relationship between EoE and food allergy is more complex. Although EoE is often considered a form of chronic, non-IgE-mediated food allergy, its underlying mechanisms differ substantially from classical IgE-mediated food allergy [159]. While genetic and environmental factors may overlap, the Th2-driven inflammatory pathways characteristic of EoE are not fully recapitulated in IgE-mediated food allergy. Consequently, the role of eosinophils in patients with concomitant EoE and food allergy remains incompletely understood and warrants further investigation.

## 8. Therapeutic Targets and Future Perspectives in EoE

Swallowed topical corticosteroids (STCs), such as budesonide and fluticasone, represent the established cornerstone of EoE therapy. Their primary mechanism is the local inhibition of type 2 inflammatory responses within the esophageal mucosa. This results in a significant reduction in eosinophil infiltration, a decrease in the release of inflammatory mediators, and improved epithelial injury and remodeling. Beyond eosinophils, corticosteroids also dampen mast cell activity and reduce the overall Th2 cytokine milieu, thereby addressing multiple cellular components of disease pathogenesis. The development of esophagus-targeted formulations has further optimized mucosal drug delivery, improving histologic remission rates while minimizing systemic absorption [13]. Biologic agents targeting IL-5 or the IL-5 receptor, including mepolizumab [160,161], reslizumab [162,163], and benralizumab [14], were designed specifically with eosinophil activity to reduce eosinophil maturation, recruitment to the esophagus, and survival in inflamed tissues, leading to consistent and sometimes profound decreases in tissue eosinophilia. However, their limited effect on symptoms and structural disease features highlights that central effector cells, eosinophils, are not the sole drivers of EoE. Accordingly, the complexity of the EoE pathogenesis described above, including both in the inflammatory and fibrotic pathways, the interplay among different inflammatory cells (e.g., eosinophils, mast cells, ILCs, etc.) and the involvement of both distinct and shared cytokine pathways (e.g., via IL-13, IL-4, IL-5), is consistent with the clinical results observed with anti-IL-5 therapies. These findings support the concept that targeting eosinophils alone may be insufficient to control disease activity and its clinical manifestations fully.

Persistent mast cell activation and upstream Th2 signaling likely contribute to ongoing inflammation and clinical manifestations despite eosinophil depletion. The IL-4 and IL-13 cytokines represent key upstream regulators of the Th2 immune response in EoE, orchestrating eosinophil recruitment, epithelial barrier dysfunction, and mast cell activation. Therapeutic blockade of this pathway, exemplified by dupilumab (anti-IL-4Rα) [123,164,165], suppresses both IL-4 and IL-13 signaling, thereby impacting multiple downstream inflammatory processes. This results in significant improvements in histologic inflammation, symptom burden, and endoscopic features. By acting at a central node of Th2 immunity, these agents simultaneously modulate eosinophil accumulation and mast cell-associated inflammatory circuits. Since mast cells are increasingly recognized as key contributors to chronic inflammation, pain perception, tissue remodeling, and smooth muscle dysfunction in EoE, novel therapeutic approaches aim to modulate mast cell function, directly or indirectly. Importantly, mast cell activity can persist even after eosinophil depletion, suggesting a partially independent role in disease maintenance. Siglec-8-targeting antibodies (e.g., lirentelimab [166]) induce eosinophil apoptosis while simultaneously inhibiting mast cell activation and degranulation [167,168]. Anti-KIT therapies, such as barzolvolimab [169], go further by targeting mast cell survival pathways, potentially reducing both mast cell-driven inflammation and secondary eosinophil recruitment. These strategies reflect a growing recognition of the functional interaction between eosinophils and mast cells in the maintenance of chronic disease.

At the highest level of the inflammatory cascade, epithelial-derived cytokines such as TSLP, IL-33, and IL-25 play a key role in initiating and amplifying Th2 immunity. These “alarmins” activate DCs and ILC2s, which in turn drive robust production of IL-5 and IL-13 [153]. This early signaling network promotes both eosinophil recruitment and mast cell activation, establishing the inflammatory loop characteristic of EoE. Therapeutic targeting of these upstream pathways (e.g., tezepelumab [170,171], solkritug [172]) aims to interrupt disease initiation and prevent downstream eosinophil- and mast cell-mediated tissue damage and remodeling. Key cytokines and therapeutic targets are summarized in Table 1.

## 9. Conclusions

EoE is a complex, chronic, immune-mediated disease in which eosinophils play a central pathogenic role, serving as key effector cells of the Th2 inflammatory response. By releasing cytotoxic granule proteins, cytokines, chemokines, and profibrotic mediators, eosinophils contribute not only to epithelial injury and barrier dysfunction but also to tissue remodeling, fibrosis, and long-term esophageal dysfunction. However, growing evidence indicates that eosinophils do not act in isolation. Instead, EoE results from a dynamic interaction between eosinophils, mast cells, Th2 lymphocytes, ILCs, DCs, and epithelial-derived cytokines, all of which cooperate to sustain chronic inflammation and disease progression. In this regard, advances in understanding EoE immunopathogenesis have profoundly transformed the therapeutic landscape. While topical corticosteroids remain highly effective in reducing eosinophilic inflammation, biologic therapies targeting IL-5, IL-4, IL-13, TSLP, Siglec-8, and mast cell pathways have highlighted the complexity of the inflammatory network underlying EoE. Importantly, the partial dissociation between histologic eosinophil depletion and clinical symptom improvement observed with some anti-IL-5 therapies suggests that persistent mast cell activation, epithelial dysfunction, and broader Th2 signaling significantly contribute to disease activity. These findings have shifted the therapeutic focus from isolated eosinophil suppression toward integrated modulation of the entire type 2 inflammatory cascade. A particularly relevant aspect emerging from recent studies is the pivotal role of esophageal remodeling and fibrosis. Beyond inflammatory activity, progressive fibrotic changes are increasingly recognized as key determinants of symptom burden, stricture formation, and long-term esophageal dysfunction. The profibrotic effects of eosinophils, together with the contribution of mast cells and epithelial–mesenchymal signaling pathways, highlight fibrosis as a central therapeutic target and an important focus for future research. Therapeutic strategies will likely shift toward precision-medicine approaches that identify distinct inflammatory endotypes and predict treatment response. In this context, therapies simultaneously targeting eosinophils, mast cells, and upstream epithelial immune pathways may offer the most effective long-term disease control. Continued translational and clinical research will be essential to better define the cellular interactions driving EoE and to develop personalized therapeutic strategies capable of achieving sustained clinical, histologic, and functional remission.

## Figures and Tables

**Table 1:** Key Cytokines, Inflammatory Mediators, and Therapeutic Targets Associated with Eosinophil-Driven Pathogenesis in Eosinophilic Esophagitis.

Cytokine/Mediator	References	Main Cellular Source	Biological Function in EoE	Therapeutic Target/Drug	Clinical Relevance
Eotaxin-3 (CCL26)	[24,26,30,104]	Esophageal epithelial cells	Major eosinophil chemoattractant induced by IL-13	Experimental CCR3 antagonists	Highly overexpressed in active EoE
Eosinophil-Derived Neurotoxin (EDN)	[133]	Activated eosinophils	Promotes tissue inflammation and immune activation	Investigational biomarker strategies	Potential biomarker of disease activity
GM-CSF	[16,66]	T cells, epithelial cells	Enhances eosinophil survival and activation	Experimental GM-CSF inhibitors	Contributes to chronic mucosal inflammation
IL-4	[17,22,23,24,25,123,164,165]	Th2 cells, basophils, mast cells	Amplifies type 2 immune responses and IgE-mediated inflammation	Dupilumab	Important in atopic comorbidities associated with EoE
IL-5	[14,43,141,142,160,161,162,163]	Th2 cells, ILC2s, mast cells	Central regulator of eosinophil differentiation, activation, and survival	Anti–IL-5: Mepolizumab; Reslizumab	Reduces tissue eosinophilia and inflammatory activity
IL-13	[24,42,48,170,171,172]	Th2 cells, ILC2s	Induces epithelial barrier dysfunction and eotaxin-3 expression	Dupilumab; cendakimab	Key driver of fibroinflammatory phenotype
IL-33	[21,72,148,154]	Epithelial and stromal cells	Alarmin cytokine promoting Th2 polarization	Anti–IL-33 therapies (investigational)	Associated with disease chronicity
Leukotrienes (LTC4, LTD4)	[128,129,130]	Eosinophils, mast cells	Potent inflammatory lipid mediators	Montelukast	Limited efficacy in EoE but relevant in allergic overlap
Major Basic Protein (MBP)	[73,74,133,134]	Activated eosinophils	Cytotoxic granule protein causing epithelial injury	No direct approved therapy	Marker of eosinophil activation
TGF-β	[59,60,61,62,63,64,126]	Eosinophils, fibroblasts, mast cells	Induces fibrosis and tissue remodeling	Anti-fibrotic approaches under investigation	Major mediator of esophageal remodeling
TSLP	[28,149,150,151,171]	Damaged epithelial cells	Activates dendritic cells and type 2 immunity	Tezepelumab (anti-TSLP, investigational)	Links epithelial injury to immune activation

## Data Availability

No new data were created or analyzed in this study.
